# Environmental Factors Associated With Preschoolers' Outdoor Play and Napping in Childcare Settings: The SUNRISE International Study

**DOI:** 10.1111/cch.70284

**Published:** 2026-05-08

**Authors:** Claudia I. Maddren, Gursimran Dhamrait, Ankhmaa Byambaa, Tawonga W. Mwase‐Vuma, Sanne L. C. Veldman, Elina Engberg, Narayan Subedi, Michael Chia, Asmaa El Hamdouchi, José Francisco López‐Gil, Fotini Venetsanou, Ijang Bih Ngyah‐Etchutambe, Clarice Maria de Lucena Martins, Chalchisa Abdeta, Najmeh Hamzavi Zarghani, Himangi Lubree, Kuston Sultoni, Pragya Singh, Edin Užičanin, Marites M. Tiongco, Mohamed‐Souhaiel Chelly, Ali Turab, Oluwayomi Abolade Aoko, Anna Kontsevaya, Jhonatan Gonzalez Santamaria, Juel Jarani, Mounir Ghogho, Anthony D. Okely

**Affiliations:** ^1^ Early Start, School of Social Sciences, Faculty of Arts, Social Sciences and Humanities University of Wollongong Wollongong NSW Australia; ^2^ Reproduction and Perinatal Centre, Westmead Hospital University of Sydney Sydney New South Wales Australia; ^3^ Centre for Social Research University of Malawi Zomba Malawi; ^4^ Mulier Institute Utrecht the Netherlands; ^5^ Folkhalsan Research Center Helsinki Finland; ^6^ Nepal Development Society Kathmandu Nepal; ^7^ Physical Education & Sports Science, National Institute of Education Nanyang Technological University Singapore; ^8^ Unite de Nutrition et Alimentation, CNESTEN Kenitra Morocco; ^9^ School of Medicine Universidad Espiritu Santo Samborondon Ecuador; ^10^ Vicerrectoria de Investigacion y Postgrado Universidad de Los Lagos Osorno Chile; ^11^ School of Physical Education and Sport Science National and Kapodistrian University of Athens Athens Greece; ^12^ University of Buea Buea Southwest Region Cameroon; ^13^ Faculty of Sports University of Porto Porto Portugal; ^14^ Early Start, School of Education, Faculty of Arts, Social Sciences and Humanities University of Wollongong Wollongong NSW Australia; ^15^ Department of Health Education and Health Promotion, Faculty of Medical Science Tarbiat Modares University Tehran Iran; ^16^ Vadu Rural Health Program King Edward Memorial Hospital Pune Maharashtra India; ^17^ Faculty of Sport and Health Education Universitas Pendidikan Indonesia Bandung Jawa Barat Indonesia; ^18^ School of Public Health and Primary Care, College of Medicine, Nursing and Health Sciences Fiji National University Suva Fiji Islands; ^19^ Faculty of Physical Education and Sport University of Tuzla Tuzla Bosnia and Herzegovina; ^20^ School of Economics De la Salle University Manila Philippines; ^21^ Sport Performance, Health & Society, Higher Institute of Sport and Physical Education of Ksar Said University of Manouba Manouba Tunisia; ^22^ Precision Health Consultants (PHC Global) Karachi Pakistan; ^23^ Human Kinetics and Health Education University of Lagos Akoka Nigeria; ^24^ National Medical Research Center for Therapy and Preventive Medicine Moscow Russia; ^25^ Fundacion Universitaria del Area Andina Pereira Risaralda Colombia; ^26^ ASSA Association Tirana Albania; ^27^ College of Computing University Mohammed VI Polytechnic Ben Guerir Rocade Rabat‐Sale Morocco; ^28^ Department of Sport, Food and Natural Sciences Western Norway University of Applied Sciences Sogndal Norway

**Keywords:** climate change, epidemiology, public health, young children

## Abstract

**Background:**

Early childhood and education centres (ECECs) are key settings in the promotion of healthy levels of outdoor play and napping among young children.

**Aim:**

This study aimed to examine the associations between environmental factors and preschoolers' outdoor play and napping in ECECs across an international sample.

**Methods:**

Data from 187 ECECs in 27 countries (22 low‐ and middle–income countries) that participated in the third pilot phase (January 2021–April 2025) of the SUNRISE International Study were analysed. The director of each ECEC completed a questionnaire which asked if children participating in the SUNRISE Study were unable to participate in outdoor play and nap time due to a range of environmental barriers.

**Results:**

Forty‐six percent (*n* = 86) of ECECs reported at least one environmental factor that prevented preschoolers' outdoor play, and 20% (*n* = 37) reported at least one factor that disrupted naptime. Hot and cold temperatures, rain and other factors were observed as barriers to outdoor play across regions and country income levels. Indoor noise, extreme temperatures, brightness and lack of space were reported as disrupting preschoolers' naptime across regions and country income levels. For rural ECECs, hot temperatures and lack of space were barriers for outdoor play and napping, respectively.

**Conclusions:**

Context‐specific strategies are required to create climate‐resilient outdoor play spaces and more restful napping environments to optimise early childhood development within ECECs.

## Summary

- Environmental conditions such as extreme temperatures, rain, noise and brightness were key barriers to outdoor play and napping in early childhood education centres (ECECs) globally.
- Strategies to improve ECEC environments must consider local context and climate to ensure children's developmental needs for play and sleep are met.
- Investment in climate‐resilient, child‐friendly infrastructure is essential to support healthy behaviours in ECEC settings, particularly in low‐ and middle–income countries.

## 1 Introduction

Approximately 60% of preschool‐aged children regularly attend an early childhood and education care service (ECEC) globally (Pre‐Primary Education. UNICEF 2020). The ECEC environment is important for the development of preschoolers' cognition, motor and social skills and language (Draper et al. 2024). ECECs can encourage participation in physical activity and outdoor play where children independently and collaboratively learn to explore their environment. Systematic reviews have found that access to outdoor environments, time spent at ECEC and teachers' education levels were positively associated with preschoolers' physical activity and outdoor play (Tonge et al. 2024; Martin et al. 2022; Lee et al. 2021). However, there is little evidence on how climate conditions (e.g., temperature and rain), and air pollution are associated with outdoor play within ECEC settings. The Children's Climate Risk Index found that approximately 1 billion children, primarily from low‐ and middle–income countries (LMICs), are exposed to extremely high‐risk climatic and environmental stressors (United Nations Children's Fund (UNICEF) 2021). Existing research, which has focused on outdoor play and physical activity within ECECs, has typically been conducted within high‐income countries (Tonge et al. 2024; Truelove et al. 2018). There is a need to better understand how environmental factors influence preschoolers' outdoor play in ECECs and how these associations may vary across different country income levels.

At ages 3 and 4, children experience a shift in their sleep patterns which in some instances leads to a cessation in daytime napping (Staton, Smith, and Thorpe 2015; Staton et al. 2020). Preschoolers attending ECECs are usually offered an opportunity to nap or to engage in quiet time, which supports the World Health Organisation (WHO) sleep recommendation of 10–13 h of good quality sleep in a 24‐h period for this age group (World Health Organization 2019). Although there is mixed evidence that supports napping in ECECs among preschool‐aged children (Staton et al. 2020; Sinclair et al. 2016; Newton and Reid 2023), there are substantial variations in ECEC provisions for naps, which are largely guided by ECEC policies, parental preference and cultural norms. A narrative synthesis by Staton, Smith, et al. (2015) found that scheduling and requirements of naps, ECEC spaces to facilitate naps and regular parental input regarding their child's participation in naps contributed to the variability in ECEC naptime. The lack of napping provisions in some ECECs may be problematic and contribute to poorer total sleep duration and quality (Staton, Irvine, et al. 2015). Although there is evidence to suggest parent perceived environmental factors such as hot and cold weather are associated with preschoolers' nighttime sleep duration (Maddren et al. 2025), there has been little consideration given to educator perceived environmental factors, which may limit opportunities for naps, particularly in LMICs. This reflects a dearth of measurement of the quality of the learning environment in LMICs (Raikes et al. 2023; Neuman and Powers 2021). As preschoolers spend a large proportion of their time within ECEC environments (Pre‐Primary Education. UNICEF 2020), it is important to understand how environmental factors may be associated with napping behaviours in these settings to ensure appropriate resources and interventions can be developed.

The aim of this study was to examine associations between environmental factors (climate, air pollution and built environment) and outdoor play and napping within ECEC settings in a diverse international sample. We explored how perceived environmental barriers varied by world region, country income level and urban/rural setting.

## 2 Methods

### 2.1 Study Design and Setting

We conducted secondary analyses of cross‐sectional data from the third pilot phase (2021–2025) of the SUNRISE International Study of Movement Behaviours in the Early Years (Okely et al. 2021) (https://sunrise‐study.com/). Human Research Ethics Approval (2018/044) was granted by the University of Wollongong, Australia. Ethics approval was also obtained by each participating country from the required institutions where applicable. A convenience cluster sampling approach was employed via ECEC services (as defined by each participating country) or, in cases where such services were not available, participants were recruited from villages, community or health care centres (Okely et al. 2021). Family day care and long day care services were not eligible for inclusion. Although preschool‐aged children are often defined as 3.0–4.99 years old, participating countries included in the sample had differing definitions. Therefore, preschool‐aged children that attended ECECs services included in this study were defined as those aged from 3.0 to 6.99 years of age who were not yet attending primary or elementary school. This study was reported using the STROBE Statement for Observational Studies (Cuschieri 2019).

### 2.2 Participants

**FIGURE 1:**
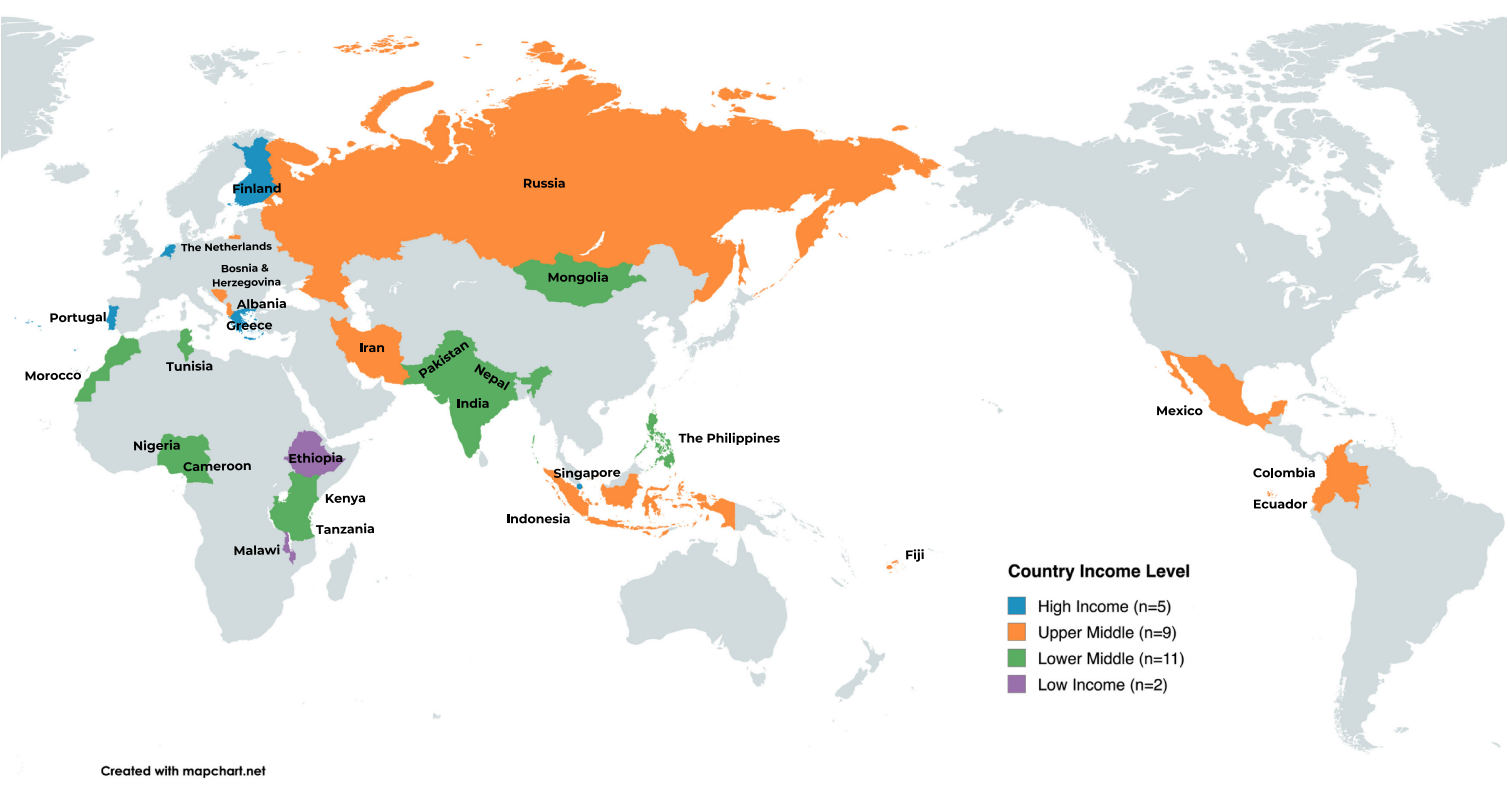
Included SUNRISE countries by country income level.

The study population included ECECs from 27 countries (Albania, Bosnia and Herzegovina, Cameroon, Colombia, Ecuador, Ethiopia, Fiji, Finland, Greece, India, Indonesia, Iran, Kenya, Malawi, Mexico, Mongolia, Morocco, Nepal, Nigeria, Pakistan, Portugal, Russia, Singapore, Tanzania, the Netherlands, the Philippines and Tunisia) (Figure 1 and Table S1). Informed consent was obtained from ECEC Directors in each participating country where applicable. ECECs with missing centre questionnaire data (*n* = 35) were excluded from the sample.

### 2.3 Measures

The director or main educator from each ECEC completed a questionnaire which asked about the ECEC environment during the third phase of SUNRISE Pilot Study data collection. The main purpose of the questionnaire was to obtain information regarding the data collection within each centre and the environmental factors that may be associated with children's 24‐h movement behaviours. Where applicable, the questionnaire was translated into local languages and completed on paper or via interview by data collectors according to the SUNRISE protocol (Okely et al. 2021). For the current study, questions which asked specifically about environmental factors that impacted participating children's outdoor play and napping behaviours were considered for analysis (See Table S2).

Educators responded to two questions regarding specific environmental factors that may have impacted on participating children's outdoor play and napping behaviours in the previous 3 days. Educators identified if heat, cold, rain, air pollution, noise and/or other factors (e.g., fear of safety [injury or kidnapping], illness and social distancing protocols) prevented children's participation in outdoor play while attending ECECs. For napping, educators reported if children's napping was disrupted due to outdoor noise, indoor noise, temperature (too hot or too cold), brightness, availability or space or other disruptions (e.g., ECEC interruptions). For both questions, educators could indicate ‘not relevant’ if no environmental barriers were perceived.

For napping, educators were asked if eligible children who were participating in the SUNRISE Study have a naptime while attending ECEC. If naps were offered, educators reported the start and end time of the naptime.

Demographic data included ECEC sector (urban/rural—defined by each participating country to ensure correct representation for each context) (Okely et al. 2021), country income level (as classified by The World Bank in the year data collection was conducted) and region (European, Latin America, Africa, Southeast Asia, Eastern Mediterranean and Western Pacific).

### 2.4 Statistical Methods

All analyses were performed in STATA 15.1 (StataCorp 2017) and jamovi [version 2.6] (Jamovi Project 2022). Descriptive statistics were used to summarise the frequency of environmental factors that influenced children's outdoor play and napping across each demographic variable. Nap duration was calculated from the difference between nap start time and nap end time. Chi‐square tests were conducted to test the association of each environmental factor with geographical region (Europe, Africa, Latin America, Southeast Asia, Eastern Mediterranean and Western Pacific), country income level (high income, upper–middle income, lower–middle income and low income) and rurality sector (urban/rural). When chi‐square assumptions were violated, Fisher's exact *p*‐value was reported (Kim 2017). The associations were considered statistically significant if the *p*‐value was < 0.05.

### 2.5 Use of Artificial Intelligence

In preparation of this work, ChaptGPT (version GPT‐4.5) by OpenAI (2025) and Claude (version 3.7 Sonnet) by Anthropic (2025) were used in the development of STATA code for analysis and manuscript editing and refinement. These tools were predominantly used to enhance clarity and readability, like an editorial review. After use, all content was reviewed and amended as required, and the authors take full responsibility for the content of this work.

## 3 Results

### 3.1 Sample Characteristics

The final sample included 187 ECECs. Sixty percent (*n* = 113) were from urban areas, whereas 40% (*n* = 74) were from rural areas. Twenty‐five percent (*n* = 46) were from high‐income countries, 33% (*n* = 62) were from upper‐middle‐income countries, 30% (*n* = 56) were from lower‐middle–income countries, and 12% (*n* = 23) were from low‐income countries. ECECs were from Europe (34%, *n* = 64), Latin America (15%, *n* = 29), Africa (24%, *n* = 44), Southeast Asia (5%, *n* = 10), Eastern Mediterranean (10%, *n* = 19) and Western Pacific (11%, *n* = 21). The distribution of ECECs by region and country income level is reported in Table S3.

### 3.2 Environmental Factors Preventing Preschoolers' Outdoor Play in ECECs

Forty‐six percent (*n* = 86) of ECECs reported at least one environmental factor that prevented preschoolers' engagement in outdoor play. Of these, rain (42%, *n* = 44), heat (23%, *n* = 24) and cold (23%, *n* = 24) were most common. Twenty‐six percent (*n* = 22) indicated other factors affected outdoor play, the most common being illness, fear of child safety and lack of outdoor play areas. Significant differences were observed between ECECs that reported at least one environmental factor as a barrier to outdoor play compared to those that did not across regions (*p* < 0.001), income level (*p* < 0.001) and sector (*p* = 0.035).

There were differences across geographical regions in the proportions reporting environmental barriers to outdoor play. Europe reported the highest proportions for cold (75%), rain (43%) and air pollution (46%), although frequencies for the latter were small (*n* = 5). Africa reported the highest proportions for heat (54%) and noise (67%); however, the frequencies for noise were small (*n* = 2). The Western‐Pacific also reported proportions between 20% and 36% for heat, rain, air pollution and noise (Table 1). The differences among geographic regions were statistically significant for heat (*p* < 0.001), cold (*p* = 0.009), rain (*p* = 0.023) and other (*p* < 0.001) (Table 2).

**TABLE 1:** Frequencies of environmental factors associated with preventing preschoolers' outdoor play in ECECs.

Predictor (*n*, %)	*n*	Not relevant (*n* = 101)	Heat (*n* = 24)	Cold (*n* = 24)	Rain (*n* = 44)	Air Pollution (*n* = 11)	Noise (*n* = 3)	Other (*n* = 22)
Region								
*EURO*	64	38 (37.6)	1 (4.2)	17 (74.5)	19 (43.2)	5 (45.5)	0 (0.0)	0 (0.0)
*LATAM*	29	20 (19.8)	0 (0.0)	1 (4.16)	5 (11.4)	1 (9.1)	0 (0.0)	2 (9.1)
*AFRO*	44	19 (18.8)	13 (54.2)	4 (16.7)	10 (22.7)	1 (9.1)	2 (66.7)	9 (40.1)
*SEARO*	10	3 (2.97)	3 (12.5)	0 (0.0)	0 (0.0)	0 (0.0)	0 (0.0)	4 (18.1)
*EMRO*	19	17 (16.8)	0 (0.0)	1 (4.2)	1 (2.3)	0 (0.0)	0 (0.0)	1 (4.5)
*WPRO*	21	4 (3.9)	7 (29.2)	1 (4.2)	9 (20.5)	4 (36.3)	1 (33.3)	6 (27.3)
Country Income								
*High income*	46	35 (34.7)	3 (12.5)	4 (16.7)	6 (13.6)	0 (0.0)	0 (0.0)	3 (13.6)
*Upper‐middle income*	62	32 (31.7)	1 (4.2)	15 (62.5)	22 (50.0)	6 (54.5)	0 (0.0)	3 (13.6)
*Lower–middle income*	56	28 (27.7)	9 (37.5)	3 (12.5)	13 (29.5)	4 (36.4)	2 (66.7)	7 (31.8)
*Low income*	23	6 (5.9)	11 (45.8)	2 (8.3)	3 (6.8)	1 (9.1)	1 (33.3)	9 (40.9)
***Sector***								
*Urban*	113	54 (53.7)	19 (79.2)	18 (75.0)	30 (68.2)	5 (45.5)	2 (66.7)	17 (77.3)
*Rural*	74	47 (46.3)	5 (20.8)	6 (25.0)	14 (31.8)	6 (54.5)	1 (33.3)	5 (22.7)

**TABLE 2:** Chi‐square test of association between environmental factors and preschoolers' outdoor play in ECECs by region, country income level and sector.

Environmental factor	Pearson chi^2^	*df*	*χ* ^2^ *p*	Fisher's exact *p*
Region				
Heat	35.84	5	—	**< 0.001**
Cold	17.29	5	—	**0.009**
Rain	12.96	5	—	**0.023**
Air pollution	10.16	5	—	0.139
Noise	5.73	5	—	0.276
Other	26.56	5	—	**< 0.0001**
**Country income level**				
Heat	34.32	3	**< 0.001**	**—**
Cold	11.01	3	**0.012**	**—**
Rain	9.14	3	**0.027**	**—**
Air pollution	4.75	3	—	0.136
Noise	4.23	3	—	0.136
Other	20.71	3	**< 0.001**	**—**
**Urban/rural**				
Heat	4.04	1	**0.044**	—
Cold	2.45	1	0.118	—
Rain	1.45	1	0.229	—
Air pollution	1.10	1	—	0.348
Noise	0.05	1	—	1.000
Other	2.96	1	0.085	—

ECECs in LMICs more frequently reported barriers to outdoor play compared with high‐income countries. Low‐income countries reported the highest proportions for heat (46%) and other environmental factors (41%). Lower‐middle–income countries reported the highest proportion for noise (67%) although frequencies were small (*n* = 2). Upper‐middle‐income countries reported the highest proportions for cold weather (63%), rain (50%) and air pollution (55%) as barriers to outdoor play in ECECs (Table 1). These differences among country income levels were statistically significant for heat (*p* < 0.001), cold (*p* = 0.012), rain (*p* = 0.027) and other (*p* < 0.001) (Table 2).

There were large differences in the presence of environmental factors that prevented outdoor play between urban and rural ECECs. Urban ECECs reported the highest proportions for heat (80%), cold (75%), rain (70%) and noise (67%) as barriers to outdoor play compared to rural ECECs. Rural ECECs reported the highest proportions for air pollution (54%) (Table 1). These differences between urban and rural ECECs were only statistically significant for heat (*p* = 0.044) (Table 3).

### 3.3 Environmental Factors Disrupting Preschoolers' Naps in ECECs

Fifty percent (*n* = 94) of ECECs indicated that children participating in the SUNRISE Pilot Study napped. Overall, educators reported a mean nap duration of 78.6 min [SD] = 34.2 min, range 14–150. ECECs located in the Western Pacific reported longer mean nap duration (118 min [SD] = 15.5 min), whereas Southeast Asian ECECs reported the shortest nap duration (45 min [SD] = 12.9 min). Educators from urban ECECs reported slightly longer nap durations among their preschoolers than those in rural ECECs (80.5 vs. 74.6 min, SD = 34.0 and 35.0, respectively). Educators from upper‐middle income countries reported a longer mean nap duration (87.3 min [SD] = 30.4 min), whereas low‐income countries reported shorter mean nap duration of 62.7 min [SD] = 19.9 min among their children. Twenty percent (*n* = 37) of ECECs reported that there was at least one environmental factor that disrupted preschoolers' naps. Significant differences were observed between ECECs that reported at least one environmental factor as a disruptor to naptime compared to those that did not across regions (*p* < 0.001), income level (*p* < 0.001), and sector (*p* = 0.001).

Although low in frequencies, there were differences across geographical regions in the proportions reporting environmental barriers to napping. Africa reported the highest proportion for indoor noise (63%), too hot (50%), lack of space (100%) and other factors (100%) that disrupted naptime. Europe had the highest proportion for outdoor noise (50%) whereas the Western Pacific ECECs reported brightness (75%) as the largest environmental factor disrupting naptime. No environmental factors were reported by ECECs that impacted preschoolers' naps in the Eastern Mediterranean, Latin America and Southeast Asia regions (Table 3). The differences among geographical regions were statistically significant for indoor noise (*p* = 0.005), too hot (p = 0.005), too cold (*p* = 0.047), brightness (*p* = 0.017) and space (*p* < 0.001) (Table 4).

**TABLE 3:** Frequencies of environmental factors disrupting preschoolers' naps in ECECs.

Predictor (*n*, %)	*n*	Not relevant (*n* = 150)	Indoor noise (*n* = 8)	Outdoor noise (*n* = 10)	Too hot (*n* = 8)	Too cold (*n* = 2)	Brightness (*n* = 4)	Space (*n* = 17)	Other (*n* = 2)
*Region*									
*EURO*	64	59 (39.33)	0 (0.0)	5 (50.0)	0 (0.0)	0 (0.0)	0 (0.0)	0 (0.0)	0 (0.0)
*LATAM*	29	29 (19.33)	0 (0.0)	0 (0.0)	0 (0.0)	0 (0.0)	0 (0.0)	0 (0.0)	0 (0.0)
*AFRO*	44	22 (14.6)	5 (62.5)	1 (10.0)	4 (50.0)	0 (0.0)	1 (25.0)	17 (100.0)	2 (100.0)
*SEARO*	10	8 (5.3)	0 (0.0)	0 (0.0)	2 (25.0)	0 (0.0)	0 (0.0)	0 (0.0)	0 (0.0)
*EMRO*	19	19 (12.7)	0 (0.0)	0 (0.0)	0 (0.0)	0 (0.0)	0 (0.0)	0 (0.0)	0 (0.0)
*WPRO*	21	13 (8.6)	3 (37.5)	4 (40.0)	2 (25.0)	2 (100.0)	3 (75.0)	0 (0.0)	0 (0.0)
*Country Income*									
*High income*	46	43 (28.7)	0 (0.0)	0 (0.0)	2 (25.0)	0 (0.0)	3 (75.0)	0 (0.0)	0 (0.0)
*Upper‐middle Income*	62	57 (38.0)	0 (0.0)	5 (50.0)	0 (0.0)	0 (0.0)	0 (0.0)	0 (0.0)	0 (0.0)
*Lower–middle Income*	56	45 (30.0)	5 (62.5)	5 (50.0)	3 (37.5)	2 (100.0)	0 (0.0)	0 (0.0)	1 (50.0)
*Low income*	23	5 (3.3)	3 (37.5)	0 (0.0)	3 (37.5)	0 (0.0)	1 (25.0)	17 (100.0)	1 (50.0)
*Sector*									
*Urban*	113	82 (54.0)	7 (87.5)	8 (80.0)	5 (62.5)	0 (0.0)	3 (75.0)	17 (100.0)	2 (100.0)
*Rural*	74	68 (46.0)	1 (12.5)	2 (20.0)	3 (37.5)	2 (100.0)	1 (25.0)	0 (0.0)	0 (0.0)

**TABLE 4:** Chi‐square test of association between environmental factors and preschoolers' naps in ECECs by region, country income level and sector.

Environmental factor	Pearson chi^2^	df	*χ* ^2^ *p*	Fisher's exact *p*
Region				
Indoor noise	15.99	5	**—**	**0.005**
Outdoor noise	12.65	5	**—**	0.061
Too hot	14.94	5	**—**	**0.005**
Too cold	15.98	5	**—**	**0.047**
Brightness	17.47	5	**—**	**0.017**
Space	60.78	5	**—**	**< 0.001**
Other	6.50	5	—	0.395
**Country income level**				
Indoor noise	12.10	3	**—**	**0.002**
Outdoor noise	6.22	3	—	0.097
Too hot	7.25	3	—	**0.033**
Too cold	4.73	3	—	0.380
Brightness	7.34	3	—	**0.022**
Space	133.34	3	**—**	**< 0.001**
Other	3.78	3	—	0.209
**Urban/rural**				
Indoor noise	2.56	1	—	0.149
Outdoor noise	1.69	1	—	0.320
Too hot	0.02	1	—	1.000
Too cold	3.09	1	—	0.155
Brightness	0.36	1	—	1.000
Space	12.25	1	**< 0.001**	**—**
Other	1.32	1	—	0.519

ECECs in low‐ and lower‐middle‐income countries more frequently reported environmental factors disrupting preschoolers' naps. Low‐income countries commonly reported too hot (38%) and lack of space (100%), although frequencies were low for the latter (*n* = 3). Lower‐middle‐income countries reported the highest proportions for indoor noise (63%) and too cold (100%, *n* = 2). Upper‐middle‐income countries reported outdoor noise (50%) as the only environmental factor to influence preschoolers' naptimes while at ECECs. High‐income countries reported the highest proportion for brightness (75%) as a disruptor to naptime (Table 3). The differences among country income levels were statistically significant for indoor noise (*p* = 0.002), too hot (*p* = 0.033), brightness (*p* = 0.022) and space (*p* = < 0.001) (Table 4).

There were large differences in the presence of environmental factors disrupting naptime between urban and rural ECECs. Urban ECECs reported the highest proportions for indoor (88%) and outdoor noise (80%), too hot (63%), brightness (75%), space (100%) and other factors (100%). Too cold was more commonly reported by rural ECECs (100%, *n* = 2) (Table 4). The differences among urban/rural areas were statistically significant for lack of space (*p* < 0.001) (Table 4).

## 4 Discussion

We examined the associations between environmental factors and preschoolers' outdoor play and napping behaviours within ECECs across an international sample. A secondary aim was to explore how environmental factors varied by world regions, country income level and urban/rural settings. Forty‐six percent of ECECs reported at least one environmental factor that prevented preschoolers from engaging in outdoor play while attending ECECs. In centres that had scheduled naptime, 20% perceived that preschoolers' naps were disrupted due to at least one environmental factor.

### 4.1 Outdoor Play

There were significant differences across geographic regions in the number of environmental factors that limited time spent in outdoor play. Compared with other regions, countries in Africa reported the highest proportion of heat and noise as a barrier to outdoor play in ECECs. The 2024 Lancet Countdown on health and climate change (Romanello et al. 2024) stated that global temperatures reached an all‐time high (1.61°C) between 2023 and 2024, exacerbating extreme heat events such as heatwaves, drought and wildfires. Such events disproportionately impact vulnerable populations like preschool‐aged children. The 2021 Children's Climate Risk Index (CCRI) (United Nations Children's Fund (UNICEF) 2021) identified that children who lived in Africa, particularly sub‐Saharan Africa, were most vulnerable to the effects of climate change, including heat. This may explain why African ECECs more commonly reported heat as a barrier to preschoolers' outdoor play. This finding may also reflect the infrastructure and resource limitations in many African ECECs. For example, limited access to electricity restricts the use of indoor cooling systems. For noise, a potential reason for our findings could be due to the location of ECECs in relation to major roads or village centres (UNESCO 2023).

Within the European region, ECECs identified cold and wet weather along with air pollution to be the most common barriers to preschoolers' outdoor play. Although colder and wetter climates are more typical in Europe compared with other regions, a potential explanation for our findings may be due to differences in parental concerns for child safety. Similar findings were observed by Kandemir and Sevimli‐Celik (2023) who found that, despite having a shared belief on the benefits of outdoor play, educators often received parental complaints when their child engaged in outdoor play during cold and wet weather due to children ending up with wet and dirty clothes, which parents perceived promoted poor hygiene and increased their risk of injury and illness. Europe and the Western Pacific reported the highest proportions of air pollution as a barrier to preschoolers' outdoor play. Although the effects of climate change and air pollution are well understood across Oceania (Asia‐Pacific Network for Early Childhood 2022), our findings for Europe were unexpected. It may be that ECEC educators within Europe have a greater understanding of the dangers of air pollution for young children, monitor air quality more regularly and keep children indoors when air quality is poor. Further, ECECs in Europe may also have policies, which provide educators with provisions for preschoolers' outdoor play when air quality is poor.

Educators from LMICs observed heat, rain, air pollution and other factors to be the main barriers to preschoolers' outdoor play. Upper‐middle income countries also identified cold weather as a barrier to outdoor play. These findings may be explained by the availability of sheltered outdoor play infrastructure (e.g., protected playground or covered outdoor learning spaces) and the provision of adequate clothing (e.g., gloves, coats, boots and hats) in more well‐resourced high‐income countries.

ECECs in urban settings consistently reported several environmental barriers to outdoor play compared to those in rural areas. Our findings could be explained by the lack of availability of space and appropriate outdoor play areas within some urban settings. Outdoor recreational spaces in high‐density urban areas are rarer, and the location of ECECs can be nested within high‐rise buildings or have limited outdoor spaces. As a greater proportion of the world's population transitions to live in urban areas, with 68% anticipated to live in metropolitan areas by 2050 (United Nations 2018), it will be necessary to consider ECECs located within urban settings to ensure preschool‐aged children can engage freely in outdoor play.

Our findings demonstrate the need for context‐specific strategies to enhance opportunities for outdoor play in ECECs in diverse settings. Investing in climate‐resilient infrastructure, educational initiatives for parents and educators, along with professional development opportunities may help minimise the perceived barriers to preschoolers' outdoor play in ECEC settings globally.

### 4.2 Napping

There were differences in the frequency of environmental factors that disrupted preschoolers' naps across geographical regions. Africa reported the highest proportion of disruptions compared with other world regions, citing indoor noise, heat and lack of space as the most common barriers to napping in ECECs. Our findings may be explained by larger class sizes in ECECs in Africa, some of whom do not participate in nap times, therefore generating noise that disturbs those children who do nap. In some instances, ECECs are located within primary schools, which may also contribute to the presence of indoor noise. Although increased temperatures continue across the world, especially in sub‐Saharan Africa (United Nations Children's Fund (UNICEF) 2021), many ECECs face significant infrastructure challenges, such as inadequate natural ventilation or limited access to cooling infrastructure such as air conditioning and fans, making nap times more uncomfortable. Further, small ECECs and classrooms may reflect the broader challenges in African early childhood education programmes, which operate in converted residential or community buildings not designed for childcare, resulting in lack of space that compromises napping among children attending ECECs (UNESCO 2023).

A small number of European and Western Pacific ECECs cited outdoor noise as a disruptor to preschoolers' naps. This may be attributed to the location of the ECECs in relation to major roads or high‐density areas. Western Pacific ECECs noted colder temperatures and brightness as disruptors to naps. Inadequate napping resources (e.g., sleeping mats, pillows, blankets and curtains) as well as poorly insulated ECECs in the Western Pacific region may explain these findings.

Educators from LMICs reported more environmental factors that disrupted napping behaviours compared to high‐income countries. Outdoor and indoor noise, hot and cold temperatures and lack of space were the most frequently reported. These findings may be explained by ECECs in LMICs having less financial support and resources to mitigate the impact of environmental factors such as extreme temperatures, inadequate ventilation or available space conducive to napping or quiet time.

Like outdoor play, urban ECECs reported higher proportions of environmental disruptors to preschoolers' nap time compared to rural ECECs. Although not specific to environmental factors or the ECECs, Zhang et al.'s (2021) systematic review on the correlates of preschoolers' sleep pattern reported that residing in urban areas was negative associated with nap duration. Our findings may be due to the location of urban ECECs in high‐density areas and closer to major roads compared with rural ECECs. This may also contribute to warmer temperatures due to buildings and concrete spaces absorbing and retaining heat compared to rural areas that typically have a higher amount of natural and greenspaces (Wald and Demorest 2022).

Despite ECECs differing in policy, resources and design, several practical elements emerged as relevant across settings. Facilitating quieter environments, using available materials such as curtains, movable mattresses and bedding to separate nap areas and adjusting daily schedules to avoid conflicts with naptimes may promote healthier napping behaviours at ECECs. In high‐resource settings, structural modifications like soundproofing or dedicated sleep rooms with adjustable lighting may be feasible. For low‐resource contexts, cost‐effective adaptations (e.g., temporary dividers and movable bedding) may provide a more restful environment.

## 5 Limitations

Our study included data from ECECs in 27 globally diverse countries, including 22 LMICs, which have limited research in this area. As data were sourced from a pilot, cross‐sectional convenience sample within each country, our findings are not generalisable and should be interpreted with caution. Our findings provide initial insights into how educators perceived environmental factors may influence preschoolers' participation in outdoor play and napping while attending ECECs. This should be further explored among larger representative samples within each included country. Data collection occurred across differing periods of the year, spanning several seasons, which made accounting for seasonality in our analyses challenging. Countries experienced varied seasons, some traditional (summer, autumn, winter and spring) and others a wet and dry season. Additionally, seasons differed substantially between countries, impacting comparability. Future research should consider objective climate data (e.g., temperature, humidity, precipitation and air pollutants) to enhance the robustness and interpretation of the findings. Only two countries comprised the low‐income country group. It is important that future research collect data from these settings as most preschool‐aged children live in LMICs and disproportionately face the threats of climate change and conflict (Parsons et al. 2025; UNICEF 2024; Abdeta et al. 2025).

## 6 Conclusions

Our study examined the associations between environmental factors and outdoor play and napping in ECECs among a geographically diverse sample of preschool‐aged children. Educators indicated hot and cold temperatures, rain and other environmental factors were barriers to outdoor play across geographic regions and country income levels. Hot temperatures were deemed a barrier in rural areas. Indoor noise, hot and cold temperatures, brightness and lack of space were observed as disrupting preschoolers' naps across regions. Indoor noise, hot temperatures, brightness and lack of space were cited as disrupting napping across country income level, whereas availability of space was a disruptor in rural areas. Researchers, policymakers and early childhood educators should consider how varying environmental factors impact access to safe outdoor play and opportunities for napping across different geographies, country income levels and urban/rural settings to enable appropriate design and implementation of interventions to promote healthy behaviours within ECECs. As urbanisation and extreme climate events continue to rise, children's health along with the environment is under threat. Future research should explore the effects of the physical and natural environment on preschoolers' opportunities to engage in outdoor play and napping while at ECECs and create strategies to mitigate their impacts.

## Author Contributions

**Claudia I. Maddren:** conceptualization, methodology, software, data curation, formal analysis, visualization, project administration, writing – original draft, writing – review and editing. **Gursimran Dhamrait:** conceptualization, supervision, writing – review and editing. **Ankhmaa Byambaa:** investigation, writing – review and editing. **Tawonga W. Mwase‐Vuma:** investigation, writing – review and editing. **Sanne L. C. Veldman:** investigation, writing – review and editing. **Elina Engberg:** investigation, writing – review and editing. **Narayan Subedi:** investigation, writing – review and editing. **Michael Chia:** investigation, writing – review and editing. **Asmaa El Hamdouchi:** investigation, writing – review and editing. **José Francisco López‐Gil:** investigation, writing – review and editing. **Fotini Venetsanou:** investigation, writing – review and editing.**Ijang Bih Ngyah‐Etchutambe:** investigation, writing – review and editing. **Clarice Maria de Lucena Martins:** investigation, writing – review and editing. **Chalchisa Abdeta:** investigation, writing – review and editing. **Najmeh Hamzavi Zarghani:** investigation, writing – review and editing. **Himangi Lubree:** investigation, writing – review and editing. **Kuston Sultoni:** investigation, writing – review and editing. **Pragya Singh:** investigation, writing – review and editing. **Edin Užičanin:** investigation, writing – review and editing. **Marites M. Tiongco:** investigation, writing – review and editing. **Mohamed‐Souhaiel Chelly:** investigation, writing – review and editing. **Ali Turab:** investigation, writing – review and editing. **Oluwayomi Abolade Aoko:** investigation, writing – review and editing. **Anna Kontsevaya:** investigation, writing – review and editing. **Jhonatan Gonzalez Santamaria:** investigation, writing – review and editing. **Juel Jarani:** investigation, writing – review and editing. **Mounir Ghogho:** investigation, writing – review and editing. **Anthony D. Okely:** conceptualization, formal analysis, funding acquisition, writing – review and editing.

## Funding

Maddren was supported by the 2022 University Postgraduate Award scholarship, through the University of Wollongong, Australia. Mwase‐Vuma was supported by Sir Halley Stewart Trust (grant number 2674). Veldman was supported by the Amsterdam Public Health Behaviours and Chronic Diseases Project Grant. Engberg was supported by the Ministry of Education and Culture, Finland and Folkhalsan. Chia was supported by the Ministry of Education, Singapore, ERFP Tier 1 (OER 04/19 TWP). El Hamdouchi was supported by the 2021 ISBNPA Pioneers Scholarship Program. Ngyah‐Etchutambe was supported by the International Society for the Study of Behavioural Development (ISSBD) Funding Program. Lubree was supported by KEM Hospital Research Centre, Pune, India. Sultoni was supported by Universitas Pendidikan Indonesia Research Grant (grant number 293/UN40‐D/PT/2019). Singh was supported by World Cancer Research Fund International and Cancer Australia. Tiongco was supported by De la Salle University, Manila, Philippines. Turab was supported by the Precision Health Consultant Internal Funding Scheme. Kontsevaya was supported by the WHO European Ooice for the Prevention and Control of Noncommunicable Diseases. Okely was supported by NHMRC Investigator Grant (GNT1175858).

## Ethics Statement

Human Research Ethics Approval (2018/044) was granted by the University of Wollongong, Australia. Ethics approval was also obtained by each participating country from the required institutions where applicable.

## Conflicts of Interest

The authors declare no conflicts of interest.

## Supporting information


**Table S1:** Sample characteristics.
**Table S2:** SUNRISE Pilot Study Centre Questionnaire—Phase III.
**Table S3:** ECEC by region and sector.

## Data Availability

The dataset used and/or analysed for the current study is available from the corresponding author on reasonable request.

## References

[cch70284-bib-0001] Abdeta, C. , D. P. Cliff , M. Toledo‐Vargas , and A. D. Okely . 2025. “24‐Hour Movement Behaviours and Health Outcomes Among Forcibly Displaced Children Affected by Conflict or Natural Disasters: A Scoping Review.” BMC Public Health 25, no. 1: 1799. 40375178 10.1186/s12889-025-22996-7 PMC12080154

[cch70284-bib-0002] Anthropic . 2025. Claude Sonnet 3.7. Claude Sonnet 3.7. https://claude.ai/new.

[cch70284-bib-0003] Asia‐Pacific Network for Early Childhood . 2022. Most Vulnerable to Most Valuable: A Scoping Study to Put Young Children at the Heart of Climate Actions and Environmental Protection. https://arnec.net/sites/default/files/2022‐12/ARNEC‐scoping%20study‐web%202022‐12‐29.pdf.

[cch70284-bib-0004] Cuschieri, S. 2019. “The STROBE Guidelines.” Saudi Journal of Anaesthesia 13, no. Suppl 1: S31–S34. 30930717 10.4103/sja.SJA_543_18 PMC6398292

[cch70284-bib-0005] Draper, C. E. , A. K. Yousafzai , D. C. McCoy , et al. 2024. “The Next 1000 Days: Building on Early Investments for the Health and Development of Young Children.” Lancet 0, no. 0: 2094–2116. 10.1016/s0140-6736(24)01389-8. PMC7617681 39571589

[cch70284-bib-0006] Jamovi Project . “Jamovi: About.” Published Online October 2022. https://www.jamovi.org/about.html.

[cch70284-bib-0007] Kandemir, M. , and S. Sevimli‐Celik . 2023. “No Muddy Shoes, No Dirty Clothes! Examining the Views of Teachers and Parents Regarding Children's Outdoor Play and Learning.” Journal of Adventure Education and Outdoor Learning 23, no. 3: 301–322.

[cch70284-bib-0008] Kim, H. Y. 2017. “Statistical Notes for Clinical Researchers: Chi‐Squared Test and Fisher's Exact Test.” Restorative Dentistry & Endodontics 42, no. 2: 152–155. 28503482 10.5395/rde.2017.42.2.152 PMC5426219

[cch70284-bib-0009] Lee, E. Y. , A. Bains , S. Hunter , et al. 2021. “Systematic Review of the Correlates of Outdoor Play and Time Among Children Aged 3‐12 Years.” International Journal of Behavioral Nutrition and Physical Activity 18, no. 1: 41. 10.1186/s12966-021-01097-9. 33736668 PMC7972019

[cch70284-bib-0010] Maddren, C. I. , G. Dhamrait , M. Ghogho , et al. 2025. “Parental Perceptions of Environmental Factors on Preschoolers' Sleep Duration Among 23 Low‐, Middle‐, and High‐Income Countries.” Behavioral Sleep Medicine Published Online October 30, 24: 1–231. 41163477 10.1080/15402002.2025.2576917

[cch70284-bib-0011] Martin, A. , R. Brophy , J. Clarke , et al. 2022. “Environmental and Practice Factors Associated With Children's Device‐Measured Physical Activity and Sedentary Time in Early Childhood Education and Care Centres: A Systematic Review.” International Journal of Behavioral Nutrition and Physical Activity 19, no. 1: 1–21. 35836231 10.1186/s12966-022-01303-2 PMC9284804

[cch70284-bib-0012] Neuman, M. J. , and S. Powers . 2021. “Political Prioritization of Early Childhood Education in Low‐ and Middle‐Income Countries.” International Journal of Educational Development 86, no. 102458: 102458.

[cch70284-bib-0013] Newton, A. T. , and G. J. Reid . 2023. “Regular, Intermittent, and Spontaneous: Patterns of Preschool Children's Nap Behavior and Their Correlates.” Sleep Medicine 102: 105–116. 36640556 10.1016/j.sleep.2022.12.019

[cch70284-bib-0014] Okely, A. D. , J. J. Reilly , M. S. Tremblay , et al. 2021. “Cross‐Sectional Examination of 24‐Hour Movement Behaviours Among 3‐ and 4‐Year‐Old Children in Urban and Rural Settings in Low‐Income, Middle‐Income and High‐Income Countries: The SUNRISE Study Protocol.” BMJ Open 11, no. 10: e049267–e049267. 10.1136/bmjopen-2021-049267 PMC8547512 34697112

[cch70284-bib-0015] OpenAI . 2025. ChatGPT 4.5. ChatGPT 4.5. https://openai.com/chatgpt/.

[cch70284-bib-0016] Parsons, E. S. , A. Jowell , E. Veidis , M. Barry , and S. T. Israni . 2025. “Climate Change and Inequality.” Pediatric Research 98: 1238–1245. 38914758 10.1038/s41390-024-03153-z PMC12549322

[cch70284-bib-0018] Raikes, A. , N. Rao , H. Yoshikawa , et al. 2023. “Global Tracking of Access and Quality in Early Childhood Care and Education.” International Journal of Child Care and Education Policy 17, no. 1: 14. 37153856 10.1186/s40723-023-00116-5 PMC10151214

[cch70284-bib-0019] Romanello, M. , M. Walawender , S. C. Hsu , et al. 2024. “The 2024 Report of the Lancet Countdown on Health and Climate Change: Facing Record‐Breaking Threats From Delayed Action.” Lancet 404, no. 10465: 1847–1896. 39488222 10.1016/S0140-6736(24)01822-1 PMC7616816

[cch70284-bib-0020] Sinclair, D. , S. Staton , S. S. Smith , C. L. Pattinson , A. Marriott , and K. Thorpe . 2016. “What Parents Want: Parent Preference Regarding Sleep for Their Preschool Child When Attending Early Care and Education.” Sleep Health 2, no. 1: 12–18. 29073446 10.1016/j.sleh.2015.11.002

[cch70284-bib-0021] StataCorp . “Stata: Release 15.” 2017.

[cch70284-bib-0022] Staton, S. , S. Irvine , C. Pattinson , S. Smith , and K. Thorpe . 2015. “The Sleeping Elephant in the Room: Practices and Policies Regarding Sleep/Rest Time in Early Childhood Education and Care.” Australasian Journal of Early Childhood 40, no. 4: 77–86.

[cch70284-bib-0023] Staton, S. , P. S. Rankin , M. Harding , et al. 2020. “Many Naps, One Nap, None: A Systematic Review and Meta‐Analysis of Napping Patterns in Children 0‐12 Years.” Sleep Medicine Reviews 50, no. 101247: 101247. 31862445 10.1016/j.smrv.2019.101247 PMC9704850

[cch70284-bib-0024] Staton, S. L. , S. S. Smith , and K. J. Thorpe . 2015. ““Do I Really Need a Nap?”: The Role of Sleep Science in Informing Sleep Practices in Early Childhood Education and Care Settings.” Translational Issues in Psychological Science 1, no. 1: 32–44.

[cch70284-bib-0025] Tonge, K. L. , M. Mavilidi , and R. A. Jones . 2024. “An Updated Systematic Review of Correlates of Children's Physical Activity and Sedentary Time in Early Childhood Education Services.” Child: Care, Health and Development 50, no. 3: e13265. 38657131 10.1111/cch.13265

[cch70284-bib-0026] Truelove, S. , B. A. Bruijns , L. M. Vanderloo , K. T. O'Brien , A. M. Johnson , and P. Tucker . 2018. “Physical Activity and Sedentary Time During Childcare Outdoor Play Sessions: A Systematic Review and Meta‐Analysis.” Preventive Medicine 108: 74–85. 29305869 10.1016/j.ypmed.2017.12.022

[cch70284-bib-0027] UNESCO . “Regional Report for Sub‐Saharan Africa : Education Starts Early; Progress, Challenges and Opportunities.” UNESCO; 2023. 10.54675/bcma5260.

[cch70284-bib-0017] UNICEF . 2020. “Pre‐Primary Education.” https://data.unicef.org/topic/education/pre‐primary‐education/.

[cch70284-bib-0028] UNICEF . 2024. “The State of the World's Children" The Future of Childhood in a Changing World.” https://www.unicef.org/media/165156/file/SOWC‐2024‐full‐report‐EN.pdf.

[cch70284-bib-0029] United Nations . 2018. 2018 Revision of World Urbanization Prospects. Accessed April 30, 2025. https://www.un.org/en/desa/2018‐revision‐world‐urbanization‐prospects.

[cch70284-bib-0030] United Nations Children's Fund (UNICEF) . 2021. The Climate Crisis Is a Child Rights Crisis: Introducing the Children's Climate Risk Index. https://www.unicef.org/media/105376/file/UNICEF‐climate‐crisis‐child‐rights‐crisis.pdf.

[cch70284-bib-0031] Wald, A. , and S. Demorest . 2022. “Race to Beat the Heat: Climate Change Impacts Physical Activity.” Journal for Nurse Practitioners 18, no. 4: 388–394.

[cch70284-bib-0032] World Health Organization . 2019. WHO Guidelines on Physical Activity, Sedentary Behaviour and Sleep for Children Under 5 Years of Age World Health Organization. https://iris.who.int/bitstream/handle/10665/311664/9789241550536‐eng.pdf?sequence=1. 31091057

[cch70284-bib-0033] Zhang, Z. , E. Sousa‐Sá , J. R. Pereira , A. D. Okely , X. Feng , and R. Santos . 2021. “Correlates of Sleep Duration in Early Childhood: A Systematic Review.” Behavioral Sleep Medicine 19: 407–425. 10.1080/15402002.2020.1772264. 32496141

