# Supplementary material for: Environmental Factors Associated With Preschoolers' Outdoor Play and Napping in Childcare Settings: The SUNRISE International Study
Source: Child Care Health Dev. 2026 May 8;52:e70284. doi: 10.1111/cch.70284 (PMC13154721; doi:10.1111/cch.70284)
Supplement: Supplementary file 1 — Table S1: Sample characteristics. Table S2: SUNRISE Pilot Study Centre Questionnaire—Phase III. Table S3: ECEC by region and sector. [file CCH-52-e70284-s001.docx]

***Table S1.*** Sample characteristics.

EURO (Europe), AFRO (Africa), LATAM (Latin America), SEARO (Southeast Asia), EMRO (Eastern Mediterranean), WPRO (Western Pacific).

|  | **n (%)** | **Urban**  **n (%)** | **Rural**  **n (%)** | **Country income level** | **Region** |
| --- | --- | --- | --- | --- | --- |
| **ECEC** | 187 (100.0) | 113 (60.43) | 74 (39.57) | - | - |
| **Countries** | | | |  |  |
| *Albania* | 8 (4.28) | 3 (2.65) | 5 (6.75) | Upper-middle income | EURO |
| *Bosnia and Herzegovina* | 8 (4.28) | 4 (3.53) | 4 (5.40) | Upper-middle income | EURO |
| *Cameroon* | 4 (2.14) | 3 (2.65) | 1 (1.35) | Lower-middle income | AFRO |
| *Colombia* | 8 (4.28) | 4 (3.53) | 4 (5.40) | Upper-middle income | LATAM |
| *Ecuador* | 11 (5.88) | 7 (6.19) | 4 (5.40) | Upper-middle income | LATAM |
| *Ethiopia* | 19 (10.16) | 19 (16.81) | 0 (0.00) | Low-income | AFRO |
| *Fiji* | 6 (3.21) | 6 (5.30) | 0 (0.00) | Upper-middle income | WPRO |
| *Finland* | 16 (8.56) | 6 (5.30) | 10 (13.5) | High-income | EURO |
| *Greece* | 14 (7.49) | 7 (6.19) | 7 (9.45) | High-income | EURO |
| *India* | 1 (0.53) | 1 (0.88) | 0 (0.00) | Lower-middle income | SEARO |
| *Indonesia* | 2 (1.07) | 2 (1.76) | 0 (0.00) | Upper-middle income | SEARO |
| *Iran* | 4 (2.14) | 4 (3.53) | 0 (0.00) | Upper-middle income | EMRO |
| *Kenya* | 5 (2.67) | 2 (1.76) | 3 (4.05) | Lower-middle income | AFRO |
| *Malawi* | 4 (2.14) | 2 (1.76) | 2 (2.70) | Low-income | AFRO |
| *Mexico* | 10 (5.35) | 7 (6.19) | 3 (4.05) | Upper-middle income | LATAM |
| *Mongolia* | 5 (2.67) | 3 (2.65) | 2 (2.70) | Lower-middle income | WPRO |
| *Morocco* | 6 (3.21) | 3 (2.65) | 3 (4.05) | Lower-middle income | EMRO |
| *Nepal* | 7 (3.74) | 2 (1.76) | 5 (6.75) | Lower-middle income | SEARO |
| *Nigeria* | 4 (2.14) | 2 (1.76) | 2 (2.70) | Lower-middle income | AFRO |
| *Pakistan* | 4 (2.14) | 2 (1.76) | 2 (2.70) | Lower-middle income | EMRO |
| *Portugal* | 4 (214) | 2 (1.76) | 2 (2.70) | High-income | EURO |
| *Russia* | 5 (2.67) | 5 (4.42) | 0 (0.00) | Upper-middle income | EURO |
| *Singapore* | 3 (1.60) | 3 (2.65) | 0 (0.00) | High-income | WPRO |
| *Tanzania* | 8 (4.28) | 3 (2.65) | 5 (6.75) | Lower-middle income | AFRO |
| *The Netherlands* | 9 (4.81) | 4 (3.53) | 5 (6.75) | High-income | EURO |
| *The Philippines* | 7 (3.74) | 4 (3.53) | 3 (4.05) | Lower-middle income | WPRO |
| *Tunisia* | 5 (2.67) | 4 (3.53) | 1 (1.35) | Lower-middle income | EMRO |

### **Table S2.** *SUNRISE Pilot Study Centre Questionnaire – Phase III*

| 8. | Do the eligible children who are participating in the SUNRISE Study have a naptime in this centre?  Yes  No | | |
| --- | --- | --- | --- |
| 8a. | If yes, what time does the nap start? (HH:MM)  ...................................................... | 8b. | What time does the nap end? (HH:MM)  ............................................ |
| 11. | In the past three days, have the eligible children who are participating in the SUNRISE Study: | | |
| 11a. | **Not** gone/been allowed to go/been taken outside to play at the because of (tick as many as appropriate) | 11b. | **Not** got enough sleep during nap time because of (tick as many as appropriate) |
|  | - Heat - Cold Rain - Air Pollution (dirty, smoky, smelly) - Noise - Other, please specify   …………………………………   - Not relevant |  | - Indoor noise - Outdoor noise (traffic/train/street noises) - Too hot - Too cold - Too much light coming into the room - Other, please specify   ………………………………………………   - Not relevant |

**Table S3.** ECEC by Region and Sector

|  | **n (%)** | **EURO** | **LATAM** | **AFRO** | **SEARO** | **EMRO** | **WPRO** |
| --- | --- | --- | --- | --- | --- | --- | --- |
| Country Income Level | | | | | | | |
| *High Income* | 46 (24.6) | 43 (67.2) | 0 (0.0) | 0 (0.0) | 0 (0.0) | 0 (0.0) | 3 (14.3) |
| *Upper-middle Income* | 62 (33.2) | 21 (32.8) | 29 (100.0) | 0 (0.0) | 2 (20.0) | 4 (21.0) | 6 (28.6) |
| *Lower-middle Income* | 56 (29.9) | 0 (0.0) | 0 (0.0) | 21 (47.7) | 8 (80.0) | 15 (78.9) | 12 (57.1) |
| *Low Income* | 23 (12.3) | 0 (0.0) | 0 (0.0) | 23 (52.3) | 0 (0.0) | 0 (0.0) | 0 (0.0) |
| Sector | | | | | | | |
| *Urban* | 113 (60.4) | 31 (48.4) | 18 (62.1) | 31 (70.5) | 4 (40.0) | 13 (68.4) | 16 (76.2) |
| *Rural* | 74 (39.6) | 33 (51.6) | 11 (37.9) | 13 (29.5) | 6 (60.0) | 6 (31.6) | 5 (23.8) |
| *Total* | 187 (100.0) | 64 (100.0) | 29 (100.0) | 44 (100.0) | 10 (100.0) | 19 (100.0) | 21 (100.0) |
